# Interleukin-1β activates matrix metalloproteinase-2 to alter lacrimal gland myoepithelial cell structure and function

**DOI:** 10.3389/fopht.2024.1415002

**Published:** 2024-06-06

**Authors:** Junji Morokuma, Angela Gárriz, Danny Toribio, Sarah Pagni, Driss Zoukhri

**Affiliations:** ^1^ Department of Comprehensive Care, Tufts University School of Dental Medicine, Boston, MA, United States; ^2^ Department of Public Health and Community Service, Tufts University School of Dental Medicine, Boston, MA, United States; ^3^ Department of Ophthalmology, Tufts University School of Medicine, Boston, MA, United States

**Keywords:** interleukin-1, lacrimal gland, myoepithelial cells, c-Jun N-terminal kinase, matrix metalloproteinase-2

## Abstract

The aim of the present study is to investigate the role of c-Jun N-terminal kinase (JNK) and matrix metalloproteinase-2 (MMP-2) in mediating the effects of interleukin-1β (IL-1β) on the function of lacrimal gland myoepithelial cells (MECs). MECs isolated from an α-smooth muscle actin–green fluorescent protein (SMA-GFP) transgenic mouse were treated with IL-1β alone or in the presence of SP600125, a JNK inhibitor, or ARP100, an MMP-2 inhibitor. The GFP intensity and the cell size/area were measured, and on day 7, the SMA, calponin, and pro-MMP-2 protein levels and the MEC contraction were assessed. At baseline, the control and treated cells showed no differences in GFP intensity or cell size. Starting on day 2 and continuing on days 4 and 7, the GFP intensity and cell size were significantly lower in the IL-1β-treated samples, and these effects were alleviated following inhibition of either JNK or MMP-2. Compared with the control, the levels of SMA and calponin were lower in the IL-1β-treated samples, and both the JNK and MMP-2 inhibitors reversed this trend. The pro-MMP-2 protein level was elevated in the IL-1β-treated samples, and this effect was abolished by the JNK inhibitor. Finally, oxytocin-induced MEC contraction was diminished in the IL-1β-treated samples, and both the JNK and MMP-2 inhibitors reversed this effect. Our data suggest that IL-1β uses the JNK/MMP-2 pathways to alter MEC functions, which might account for the diminished tears associated with aqueous-deficient dry eye disease.

## 1 Introduction

The lacrimal gland is an exocrine gland whose main function is to synthesize, store, and secrete proteins along with electrolytes and water onto the ocular surface (1–3). Secretions from the lacrimal gland are the major components of the aqueous layer of the preocular tear film, and many of these secreted proteins have antimicrobial or growth factor properties that help protect and nourish the non-keratinized ocular surface epithelia (1, 2). The lacrimal gland is composed primarily of acinar cells, which synthesize and secrete the primary fluid; ductal cells, which modify the electrolyte composition of the primary fluid; and myoepithelial cells (MECs), which surround acini and ducts and contract, upon stimulation, to help expel the lacrimal gland fluid onto the ocular surface (2, 4, 5).

In recent studies, it has been reported that lacrimal gland MECs express oxytocin (OXT) and purinergic and cholinergic M_3_ muscarinic receptors (6–8). It is now well documented that lacrimal gland MECs can respond and contract in response to various stimuli, such as OXT and cholinergic and purinergic agonists, which are also known to cause lacrimal gland secretion (6, 8–10). It appears therefore that MEC, in response to neural stimuli, act in tandem with the acinar and ductal cells to ensure adequate secretion of the lacrimal gland fluid onto the ocular surface. Our previous studies showed that the oxytocin receptor (OXTR) uses multiple signaling pathways to stimulate MEC contraction and that the expression (and therefore function) of this receptor is downregulated in chronically inflamed lacrimal glands (6, 10, 11). These findings suggest that OXT/OXTR signaling might play a prominent role in modulating the function of lacrimal gland MECs akin to its well-established role in controlling mammary gland MECs.

Chronic inflammation of the lacrimal gland, as occurs in Sjogren’s syndrome, is a leading cause of aqueous-deficient dry eye disease and affects an estimated 1–4 million North Americans, mostly women (12, 13). The mechanisms leading to insufficient lacrimal gland secretion due to chronic inflammation are still not completely understood, hence the lack of treatment modalities (14, 15). In previous studies, we reported on the pivotal role that pro-inflammatory cytokines, especially interleukin-1β (IL-1β), play in the deregulated lacrimal gland secretion. We showed that IL-1β inhibited the release of neurotransmitters from lacrimal gland nerve endings, both *in vitro* and *in vivo*, leading to impaired lacrimal gland secretion (16–18). Recently, we have reported that chronic treatment of lacrimal gland MECs with IL-1β resulted in the degradation of two contractile proteins, α-smooth muscle actin (SMA) and calponin, which led to impaired MEC contraction in response to OXT stimulation (19). These *in vitro* findings mimicked the *in vivo* findings we reported using animal models of Sjogren’s syndrome dry eye disease, namely, a reduction in MEC size, the degradation of contractile proteins, and the impairment of MEC contraction in response to OXT stimulation (6).

It is well documented that IL-1β can activate c-Jun N-terminal kinase (JNK) (20, 21). Previously published work from our laboratory supports a role for JNK activity in the IL-1β-mediated inhibition of lacrimal gland secretion (22). JNK activation is also known to lead to matrix metalloproteinase-2 (MMP-2) activation in several tissues (23–29). In a recent study, we have shown that MMP-2 activity was increased in chronically inflamed lacrimal glands (30). Furthermore, we reported that the inhibition of either JNK or MMP-2 activity restored tear secretion in animal models of Sjogren’s syndrome dry eye disease (22, 30). Therefore, in the present study, we aimed to examine the potential role of JNK and MMP-2 in mediating the effects of IL-1β on the structure and function of lacrimal gland MECs.

## 2 Materials and methods

### 2.1 Chemicals and antibodies

Recombinant human IL-1β was purchased from PEPROTECH (Rocky Hill, NJ, USA). Oxytocin (O3215) was purchased from Sigma-Aldrich (Saint Louis, MO, USA). The broad-spectrum JNK inhibitor, anthra[1–9-*cd*]pyrazol-6(2*H*)-one (SP600125) (11), and the MMP-2 selective inhibitor, *N*-hydroxy-2-[(4-phenylphenyl)sulfonyl-propan-2-yloxyamino]acetamide (ARP100) (31), were purchased from Bio-Techne Corporation (Minneapolis, MN, USA). The cell media Dulbecco’s modified Eagle’s medium (DMEM) and RPMI-1640 medium (Roswell Park Memorial Institute), collagenase type II, penicillin–streptomycin, l-glutamine, TrypLE Express, and fetal bovine serum (FBS) were from Thermo Fisher Scientific (Waltham, MA, USA). The mouse monoclonal antibody against MMP-2 (ab86607 at 1:500 dilution), the rabbit polyclonal antibody against αSMA (ab5694 at 1:400 dilution), and the rabbit monoclonal antibody against calponin (ab46794 at 1:2,000 dilution) were from Abcam (Waltham, MA, USA). The rabbit monoclonal antibody against β-actin (clone HL1926, 1:10,000 dilution) was from GeneTex (Irvine, CA, USA). The IRDye-labeled secondary antibodies were from LI-COR (Lincoln, NE, USA) and were used at a 1:5,000 dilution.

### 2.2 Animals

All experiments were performed in accordance with the Association for Research in Vision and Ophthalmology (ARVO) statement for the use of animals in ophthalmic and vision research and were approved by the Tufts Medical Center Institutional Animal Care and Use Committee. Mice were maintained in rooms of constant temperature with fixed light/dark intervals of 12-h length and were fed *ad libitum*. To obtain MECs, the αSMA-GFP mouse strain (C57BL6/SMACreErt2) described by Yokota et al. (32) was used for this study, a kind gift from Dr. Ivo Kalajzic (33) (UConn Health, Farmington, CT, USA). In these mice, the lacrimal gland MECs that express SMA were therefore labeled with GFP and can be easily identified in the culture dishes.

### 2.3 Isolation and propagation of lacrimal gland MECs

Lacrimal gland MECs were isolated as previously described (10, 19). Briefly, 4- to 6-week-old SMA-GFP mice were euthanized and the exorbital lacrimal glands were removed, washed in cold DMEM, gently minced with a scalpel and forceps to prepare 2- to 3-mm lobules, and placed in digestion media (1.5 mL/gland of DMEM containing 1.65 mg/mL of collagenase type II). The samples were then incubated in a shaking water bath (37°C and 100 rpm) for 20–30 min. At regular 5-min intervals, the lobules were gently pipetted 10 times through tips of decreasing diameter. The digested tissue was filtered through a sterile cell strainer (100-μm nylon mesh; Thermo Fisher Scientific, Waltham, MA, USA). The cells were washed with 1–2 mL DMEM and then centrifuged at 100 × *g* for 5 min. The cells were resuspended in complete RPMI-1640 medium supplemented with 10% FBS, 2 mM l-glutamine, and 100 μg/mL penicillin–streptomycin, plated in 100-mm culture dishes (VWR, Radnor, PA, USA), and placed in a 37°C incubator (5% CO_2_).

### 2.4 Cytokine treatment of lacrimal gland MECs

Lacrimal gland MECs were seeded in six-well plates in RPMI medium containing 10% FBS. Confluent to sub-confluent MEC cultures were either left untreated or were treated with 10 ng/mL of IL-1β for a total of 7 days, as previously described (19). Select wells received the JNK inhibitor SP600125 (20 µM) or the MMP-2 inhibitor ARP100 (10 µM) 30 min prior to the addition of IL-1β. The media (containing fresh IL-1β, SP600125, or ARP100) were changed every other day, and four to five photographs were randomly taken from each well to measure the GFP intensity and cell size. After the last day of treatment (day 7), the cells were either lysed and the protein samples prepared for Western blotting or were trypsinized to perform contraction experiments, as described below.

### 2.5 Image analysis of GFP intensity and cell size measurements

Measurements of the GFP intensity and cell size were performed as previously described (19). Briefly, still images of the MEC cultures were taken on day 0 (before the addition of cytokines/inhibitors) and then at 2, 4, and 7 days of IL-1β ± JNK or MMP-2 inhibitor treatment using a digital camera (SPOT Insight CMOS; SPOT Imaging, Sterling Heights, MI, USA) mounted on an inverted light microscope (Eclipse TE2000-S; Nikon Instruments Inc., Melville, NY, USA). The total GFP intensity was analyzed from the control and treated MECs using ImageJ/Fiji software (ImageJ 1.54f; National Institutes of Health, Bethesda, MD, USA). Measurement of the GFP intensity is an indirect indicator of the level of SMA protein as the GFP expression in MECs is controlled by the SMA promoter. In addition, a minimum of five to eight cells per photograph were selected to measure the cell area (in square micrometers) using the SPOT Advanced Imaging software (version 5.6).

### 2.6 SDS-PAGE and Western blotting

At the end of the cytokine treatment, lacrimal gland MECs from the treated and control groups were lysed in 0.2 mL ice-cold radioimmunoprecipitation assay (RIPA) buffer [10 mM Tris–HCl (pH 7.4), 150 mM NaCl, 1 mM EDTA, 1% Triton X-100, 0.1% sodium deoxycholate, and 0.1% SDS supplemented with protease inhibitors], as previously described (19). The cell lysates were centrifuged at 20,000 × *g* for 30 min and the supernatant collected. The proteins were separated by SDS-PAGE on NuPage 4%–12% Bis-Tris gels in MOPS-SDS buffer (Thermo Fisher Scientific, Waltham, MA, USA). The proteins in the gels were transferred to PVDF membranes using the NuPage transfer buffer (Thermo Fisher Scientific, Waltham, MA, USA) and processed for immunoblotting. After transfer, the PVDF membranes were stained with the REVERT Total Protein Stain (LI-COR, Lincoln, NE, USA), following the manufacturer’s instructions, and prior to being blocked using the Odyssey blocking buffer (LI-COR, Lincoln, NE, USA) for 1 h at room temperature. The membranes were then incubated overnight at 4°C with the appropriate primary antibodies diluted in a blocking buffer. Following washing with Tris-buffered saline + Tween-20 (TBST; 50 mM Tris–HCl, 150 mM NaCl, and 0.1% Tween-20, pH 7.6), the membranes were then incubated for 1 h at room temperature with the appropriate secondary antibodies, followed by detection on a LI-COR Odyssey Infrared Imager. The REVERT Total Protein Stain from each lane, and the immunoblotted band intensity, was quantified using the LI-COR Image studio software (v.5.2). Western blot band quantifications were then normalized to the total amount of protein in each lane or to the total amount of β-actin for the MMP-2 blots.

### 2.7 MEC contraction assay

After 7 days of IL-1β ± JNK or MMP-2 inhibitor treatment, the cells were trypsinized and seeded overnight in a 24-well-plate at a density of around 5,000 cells/well. The cells treated with IL-1β and IL-1β ± JNK or MMP-2 inhibitors and the untreated cells (control) were stimulated with OXT (10^−6^ M) for 20 min. Still images of the contraction process were taken using a digital camera (SPOT Insight CMOS; SPOT Imaging, Sterling Heights, MI, USA) mounted on an inverted fluorescence microscope (Eclipse TE2000-S; Nikon Instruments Inc., Melville, NY, USA), as previously described (19). The MEC images were taken at time 0 and 10 min before stimulation and then 20 min after OXT stimulation and were used for image analyses. At least 10 random cells from each well and each condition were used for image analyses using ImageJ/Fiji software (ImageJ 1.54f; National Institutes of Health, Bethesda, MD, USA). The perimeter of the same cell was calculated before and after OXT stimulation, and the difference between these two values that represents the decrease in cell size after OXT stimulation was expressed as percentage change.

### 2.8 Statistical analyses and data presentation

Statistical analyses were performed using GraphPad Prism software (version 10.0; San Diego, CA, USA). Where appropriate, data are presented as the mean ± standard deviation (SD). Normality and log-normality tests were performed to determine whether the data followed a normal (Gaussian) distribution. Data were analyzed using one-way analysis of variance (ANOVA), followed by Tukey’s multiple comparison tests for normally distributed data or with the Kruskal–Wallis followed by Dunn’s multiple comparisons tests for non-normally distributed data. Statistically significant differences were considered at a *p*-value <0.05.

## 3 Results

### 3.1 Role of JNK in the effects of IL-1β on GFP intensity and MEC size

**Figure 1:**
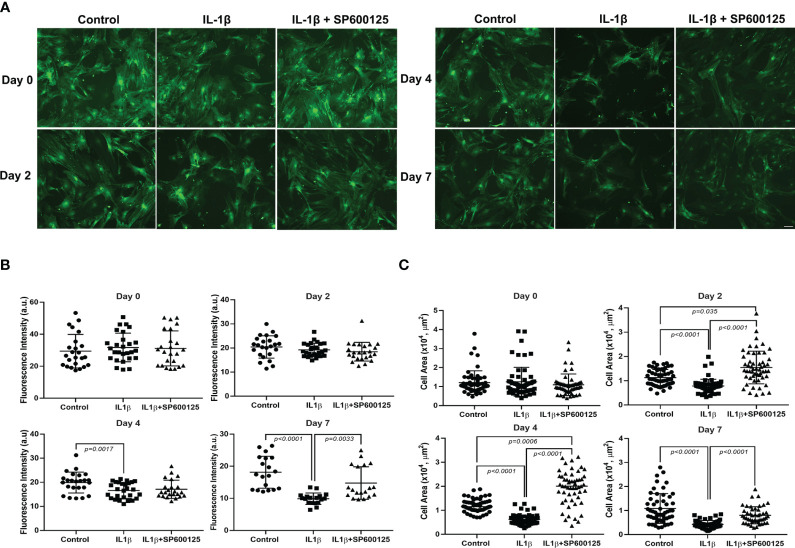
Effect of interleukin-1β (IL-1β) ± c-Jun N-terminal kinase (JNK) inhibitor SP600125 on green fluorescent protein (GFP) intensity and myoepithelial cell (MEC) size. Lacrimal gland MECs were either left untreated (control) or incubated with IL-1β (10 ng/mL) alone or with IL-1β plus the JNK inhibitor SP600125 (20 µM) for 2, 4, and 7 days. Up to 10 random images were taken using the same camera setting and were used to quantify the GFP intensity and MEC size. The GFP intensity was measured using the ImageJ/Fiji software, while the MEC size was quantified using SPOT Imaging software, as described in *Materials and methods*. **(A)** Representative photomicrographs depicting the effects of IL-1β ± JNK inhibitor on MECs. Similar results were obtained in at least four independent experiments. *Scale bar*, 100 µm. **(B)** Compared with the control group, IL-1β treatment significantly decreased the intensity of GFP and JNK inhibition significantly rescued the effect of IL-1β treatment over time. Data from five independent experiments are presented as the mean ± SD. *n* = 24–27 for days 0 and 2; *n* = 23–27 for day 4; and *n* = 18–19 for day 7. **(C)** Compared with the control group, IL-1β treatment significantly decreased the size of MECs and JNK inhibition significantly rescued the effect of IL-1β treatment over time. Data are from four independent experiments presented as the mean ± SD. *n* = 54–63 for days 0, 2, and 4; *n* = 47–60 for day 7.

Lacrimal gland MECs were incubated with IL-1β in the presence or absence of the broad-spectrum JNK inhibitor SP600125 (11), and the GFP intensity and cell size were measured at baseline (day 0) and then 2, 4, and 7 days later. As shown in **Figure 1A**, on day 0, the GFP intensities were similar in the control and the IL-1β- and IL-1β + SP600125-treated cells. Compared with that in untreated control cells, the GFP intensity in the IL-1β-treated samples started to decline, reaching a maximum decrease of 54% by day 7 (**Figure 1B**). SP600125 significantly alleviated the inhibitory effect of IL-1β on GFP intensity from 54% to 16% (**Figure 1B**). At baseline, the MEC sizes were similar in the control and the IL-1β- and IL-1β + SP600125-treated cells (**Figure 1C**). In contrast, starting on day 2 and continuing to day 7, the MEC size significantly decreased in the IL-1β-treated group (a 61% decrease relative to the control) (**Figure 1C**). SP600125 significantly alleviated the inhibitory effect of IL-1β on MEC size from 61% to 26% on day 7 (**Figure 1C**). Similarly, SP600125 treatment significantly alleviated the inhibitory effect of IL-1β on days 2 and 4 (**Figure 1C**).

These data suggest that the JNK pathway is activated by IL-1β to alter the morphology of MECs by reducing the GFP intensity (SMA expression), resulting in reduced cell size.

### 3.2 Role of MMP-2 in the effects of IL-1β on GFP intensity and MEC size

**Figure 2:**
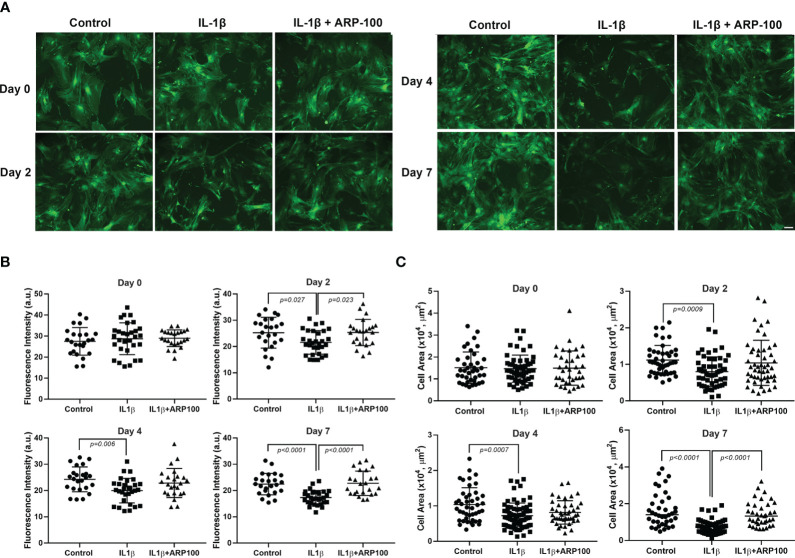
Effect of interleukin-1β (IL-1β) ± matrix metalloproteinase-2 (MMP-2) inhibitor ARP100 on green fluorescent protein (GFP) intensity and myoepithelial cell (MEC) size. Lacrimal gland MECs were either left untreated (control) or incubated with IL-1β (10 ng/ml) alone or with IL-1β plus the MMP-2 inhibitor ARP100 (10 µM) for 2, 4, or 7 days. Up to 10 random images were taken using the same camera setting and were used to quantify the GFP intensity and MEC size. The GFP intensity was measured using ImageJ/Fiji software, while the MEC size was quantified using SPOT Imaging software, as described in the *Materials and methods*. **(A)** Representative photomicrographs depicting the effects of IL-1β ± MMP-2 inhibitor on MECs. Similar results were obtained in at least four independent experiments. *Scale bar*, 100 µm. **(B)** Compared with the control group, IL-1β treatment significantly decreased the GFP intensity and MMP-2 inhibition significantly rescued the effect of IL-1β treatment over time. Data from four independent experiments are presented as the mean ± SD. *n* = 24–30 for all days. **(C)** Compared with the control group, IL-1β treatment significantly decreased the MEC size and MMP-2 inhibition significantly rescued the effect of IL-1β treatment over time. Data are from three independent experiments presented as the mean ± SD. *n* = 37–55 for day 0; *n* = 45–54 for day 2; *n* = 43–74 for day 4; *n* = 37–57 for day 7.

In another series of experiments, we examined the effect of the selective MMP-2 inhibitor, ARP100 (31), on the IL-1β-induced decrease in GFP intensity and MEC size. On day 0, the GFP intensities were similar in the control and the IL-1β- and IL-1β + ARP100-treated cells (**Figure 2A**). Starting on day 2 and continuing onto day 7, the GFP intensity was significantly lower in IL-1β-treated cells (**Figure 2B**). Pretreatment of the cells with ARP100 significantly and completely alleviated the effect of IL-1β on GFP intensity on day 7 (compared with the control, the GFP intensity was down by 22% on day 7, but 0% with the MMP-2 inhibitor) (**Figure 2B**). We subsequently examined the effect of the MMP-2 inhibitor on the IL-1β-induced reduction in MEC size. As shown in **Figure 2C**, on day 0, the MEC sizes were similar in the control and the IL-1β- and IL-1β + ARP100-treated cells. The MEC size significantly decreased following IL-1β treatments, with a maximum 54% decrease by day 7 (**Figure 2C**). The inhibition of MMP-2 by ARP100 significantly alleviated the effect of IL-1β on MEC size from 54% to 13% (**Figure 2C**).

**Figure 3:**
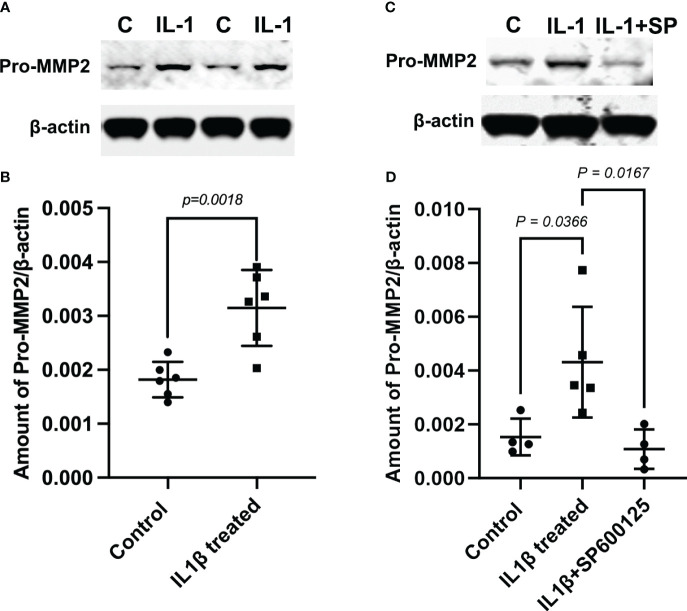
Role of c-Jun N-terminal kinase (JNK) in the interleukin-1b (IL-1b)-induced increase of matrix metalloproteinase-2 (MMP-2) protein expression. Lysates were prepared from control myoepithelial cells (MECs) and from MECs treated with IL-1β- and IL-1β plus the JNK inhibitor SP600125. Samples were subjected to SDS-PAGE followed by Western blotting using a monoclonal anti-MMP-2 antibody or a polyclonal anti-β-actin antibody, used as a loading control. **(A, C)** Representative blots. **(B, D)** Data from four to six independent experiments are presented as the mean ± SD.

In addition, we performed Western blotting experiments to determine whether IL-1β can activate MMP-2 in MECs. The generation of active MMP-2 (~64 kDa) occurs via the proteolysis of the pro-MMP-2 (~72 kDa) protein by membrane type 1 matrix metalloproteinase (MT1-MMP, MMP14) (34–36). We used a monoclonal antibody that recognizes both the pro- and active forms of MMP-2 on lysates from the control and IL-1β-treated MEC samples. As shown in **Figures 3A, B** (and **Supplementary Figure S1**), compared with untreated control MECs, there was a 1.73-fold increase in the amount of pro-MMP-2 protein in IL-1β-treated MECs. However, the active MMP-2 could not be detected with this antibody or with another monoclonal antibody from Novus Biologicals. Since JNK activation is known to lead to MMP-2 activation in several tissues, we examined the effect of the JNK inhibitor, SP600125, in IL-1β-treated cells. As shown in **Figures 3C, D** (and **Supplementary Figure S1**), the IL-1β-induced increase in pro-MMP-2 protein was completely blocked by SP600125.

Collectively, these data suggest that IL-1β activates MMP-2 via the JNK pathway, which leads to alterations in the morphology of MECs by reducing the GFP intensity (SMA expression), resulting in reduced cell size.

### 3.3 Role of JNK and MMP-2 in the effects of IL-1β on MEC contractile protein expression

**Figure 4:**
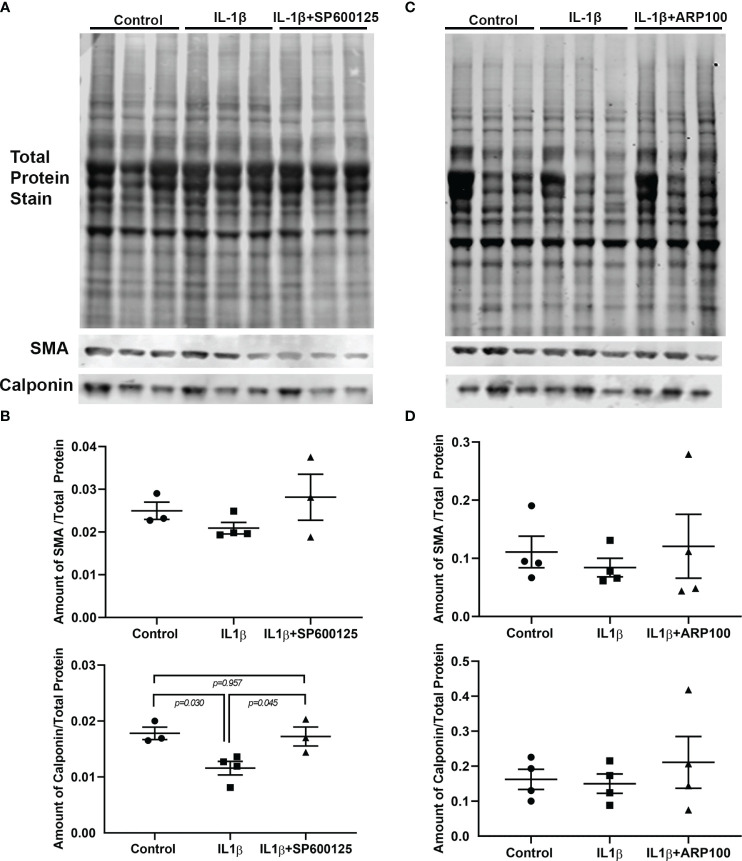
Effect of interleukin-1β (IL-1β) ± c-Jun N-terminal kinase (JNK) inhibitor SP600125 and IL-1β ± matrix metalloproteinase-2 (MMP-2) inhibitor ARP100 on α-smooth muscle actin (SMA) and calponin protein expression levels. Lacrimal gland myoepithelial cells (MECs) were either left untreated (control) or incubated with IL-1β (10 ng/ml) alone, with IL-1β plus the JNK inhibitor SP600125 (20 µM), or with IL-1β plus the MMP-2 inhibitor ARP100 (10 µM) for 7 days. The SMA and calponin protein levels were quantified by Western blotting and reported as a ratio relative to the total protein stain, as described in *Materials and methods*. **(A, C)** Representative images of total protein stain and Western blots for SMA and calponin. **(B, D)** Plots showing the levels of SMA and calponin in the control and treated lacrimal gland MECs relative to the total protein stain. **(B)** The level of SMA protein tended to decrease with IL-1β treatment and was rescued by JNK inhibition, although not statistically significantly. Compared with the control group, IL-1β treatment significantly decreased the level of calponin and JNK inhibition significantly rescued the effect of IL-1β. Averaged data from three to four independent experiments are presented as the mean ± SEM (*n* = 3–4 in each experimental group). **(D)** Plot showing the levels of SMA and calponin in the control and treated lacrimal gland MECs relative to the total protein stain. The levels of both proteins tended to decrease with IL-1β treatment and was rescued by MMP-2 inhibition, although not statistically significantly. Averaged data from four independent experiments are presented as the mean ± SEM (*n* = 7 in each experimental group).

We previously reported that chronic treatment of lacrimal gland MECs with IL-1β led to the lower expression of two contractile proteins, i.e., SMA and calponin, most likely through the initiation of a proteolytic process (19). In the next series of experiments, we investigated whether either JNK or MMP-2 are involved in this process. As shown in **Figures 4A, B** (and **Supplementary Figure S2**), the levels of SMA and calponin were lower, although not statistically significantly for SMA, in IL-1β-treated MECs. Importantly, the inhibition of JNK by SP600125 completely alleviated the effects of IL-1β on the SMA and calponin levels (**Figure 4**). Similar results were obtained when the MMP-2 inhibitor ARP100 was used, i.e., a trend of lower expression of SMA and calponin in IL-1β-treated samples, which was reversed by the MMP-2 inhibitor ARP100 (**Figures 4C, D** and **Supplementary Figure S3**).

These data suggest that both JNK and MMP-2 pathways are involved in the IL-1β-induced degradation of SMA and calponin in lacrimal gland MECs.

To determine whether the so far reported effects of IL-1β on the GFP intensity, cell size, and SMA and calponin levels of MECs were due to IL-1β inhibiting cell proliferation or inducing cell death, we quantified all the total protein-stained membranes from the control, IL-1β, and IL-1β plus inhibitor groups. The expectations were that, if IL-1β was inhibiting cell proliferation or inducing cell death, then the total amount of protein from the IL-1β-treated samples should be lower when compared with that from untreated control MECs. As shown in **Supplementary Figure 4**, this was not the case, as the total amount of protein from the IL-1β-treated samples was almost identical to that from control MECs. Similarly, the total amount of protein in the samples treated with IL-1β plus inhibitor was also comparable to that from the control samples.

### 3.4 Role of JNK and MMP-2 in the effects of IL-1β on OXT-induced MEC contraction

**Figure 5:**
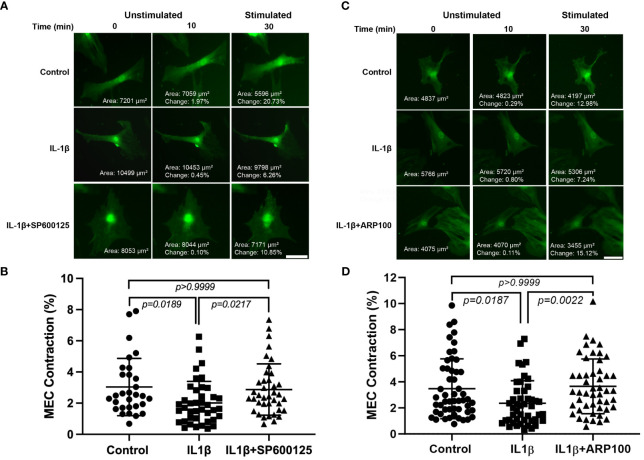
Effect of interleukin-1β (IL-1β) ± c-Jun N-terminal kinase (JNK) inhibitor SP600125 and IL-1β ± matrix metalloproteinase-2 (MMP-2) inhibitor ARP100 on oxytocin (OXT)-induced lacrimal gland myoepithelial cell (MEC) contraction. Lacrimal gland MECs were either left untreated (control) or incubated with IL-1β (10 ng/ml) alone, IL-1β plus the JNK inhibitor SP600125 (20 µM), or IL-1β plus the MMP-2 inhibitor ARP100 (10 µM) for 7 days. The cells were trypsinized, reseeded at low density, and incubated overnight. They were then stimulated with OXT (10 µM) for 20 min and the changes in MEC size (i.e., contraction) following OXT stimulation were measured using ImageJ/Fiji software, as described in *Materials and methods*. **(A, B)** Chronic treatment of MECs with IL-1β significantly inhibited the OXT-induced contraction, and the inhibition of JNK significantly rescued the effect of IL-1β. Data from three independent experiments are presented as the mean ± SD (*n* = 30–40). *Scale bars*, 20 µm. **(C, D)** Chronic treatment of MECs with IL-1β significantly inhibited the OXT-induced contraction, and inhibition of MMP-2 significantly rescued the effect of IL-1β. Data from three independent experiments are presented as the mean ± SD (*n* = 46–52). All *scale bars*, 20 µm.

In our previous work, we reported that chronic treatment of lacrimal gland MECs with IL-1β led to the inhibition of OXT-induced lacrimal gland MEC contraction (19). In the next series of experiments, we used the JNK and MMP-2 inhibitors to determine whether either pathway is involved in the IL-1β-induced inhibition of OXT-induced MEC contraction. As shown in **Figures 5A, B**, compared with the control, the OXT-induced MEC contraction in IL-1β-treated samples was inhibited by 33.6%. Pre-incubation of the cells with the JNK inhibitor SP600125 almost completely reversed the inhibitory effect of IL-1β on the OXT-induced MEC contraction (down from 33.6% in the IL-1β group, to 5.2% in the IL-1β + SP600125 group, when compared with the control) (**Figure 5B**), suggesting that the JNK pathway is involved in this process. Similarly, the diminished response to the OXT-stimulated MEC contraction in IL-1b-treated samples (32% down when compared with the control) (**Figures 5C, D**) was completely reversed when the cells were incubated with the MMP-2 inhibitor ARP100 (5.2% higher when compared with the control) (**Figure 5D**).

These data suggest that both JNK and MMP-2 pathways are involved in the IL-1β-induced inhibition of OXT-induced MEC contraction.

## 4 Discussion

In previous studies, we reported that chronic treatment of lacrimal gland MECs with IL-1β led to the degradation of contractile proteins, which resulted in reduced MEC size and inhibition of the OXT-induced contraction (19). Other studies from our group showed that the MECs of chronically inflamed lacrimal glands of animal models of Sjogren’s syndrome dry eye disease had reduced size, decreased contractile protein expression, and loss of contraction in response to OXT stimulation (6). In the present study, we showed that JNK and MMP-2 are pivotal mediators of IL-1β activity on the structure and function of lacrimal gland MECs.

JNK activation is known to lead to MMP-2 activation in several tissues (23–29). The activation of MMP-2 occurs via proteolysis of the pro-MMP-2 protein and involves MT1-MMP (MMP14) and tissue inhibitor of metalloproteinases 2 (TIMP-2) (34–36). Active MT1-MMP anchored on the cell surface acts as a receptor for TIMP-2, and this binary complex acts as a receptor for pro-MMP-2 (34, 35, 37, 38). MT1-MMP then cleaves pro-MMP-2, generating an intermediate species with further autocatalytic proteolysis generating the fully active MMP-2 (34, 35). Several studies have shown that the activated JNK led to the activation/increased expression of both MT1-MMP and TIMP2, thus activating MMP-2 (23, 25).

The activation of JNK in quiescent microvascular endothelial cells was sufficient to induce the expression of both MMP-2 and MT1-MMP mRNA, which resulted in an increased MMP-2 production and activation (23). The downregulation of c-Jun, a downstream target of JNK, with small interference RNA inhibited the expression of MMP-2 in stimulated endothelial cells (23). In C6 rat glioma cells, it was shown that JNK activity controlled the expression of both MT1-MMP and TIMP-2, which led to the activation of MMP-2 in these cells (25).

Several studies have also reported that MMP-2 activation led to the degradation of calponin in vascular smooth muscle cells (39–41). MMP-2 and calponin were shown to colocalize within the cells and can be co-immunoprecipitated (39–41). Furthermore, incubation of calponin with MMP-2, *in vitro*, led to the proteolysis of calponin, an effect that was blocked by GM6001, an MMP inhibitor (40). In cardiac myocytes, MMP-2 activation has been linked to the proteolysis of several contractile proteins including troponin I (42), myosin light chain-1 (43), α-actinin (44), and titin (45), thereby reducing contractile function. MMP-2 activity has also been linked to SMA degradation in several tissues (46). In vascular smooth muscle cells, it was demonstrated that both platelet-derived growth factor (PDGF) and insulin-like growth factor (IGF-I) stimulation resulted in an increased MMP-2 expression, which led to a decreased SMA expression (47). In a uremic mouse model of chronic kidney disease, a vascular smooth muscle cell phenotype change, reflected by the loss of SMA and other smooth muscle markers and the upregulation of MMP-2, preceded cell loss and arterial medial calcification (48, 49). In 2D culture systems, neonatal rat cardiomyocytes underwent reversible rearrangement of their contractile apparatus, with the inherent α-cardiac actin isoform transiently replaced by SMA and the loss of contractility (50). After several days in culture, the degradation of SMA, following the upregulation of MMP-2 expression, paralleled the restoration of the myofibrillar system (50, 51).

**Figure 6:**
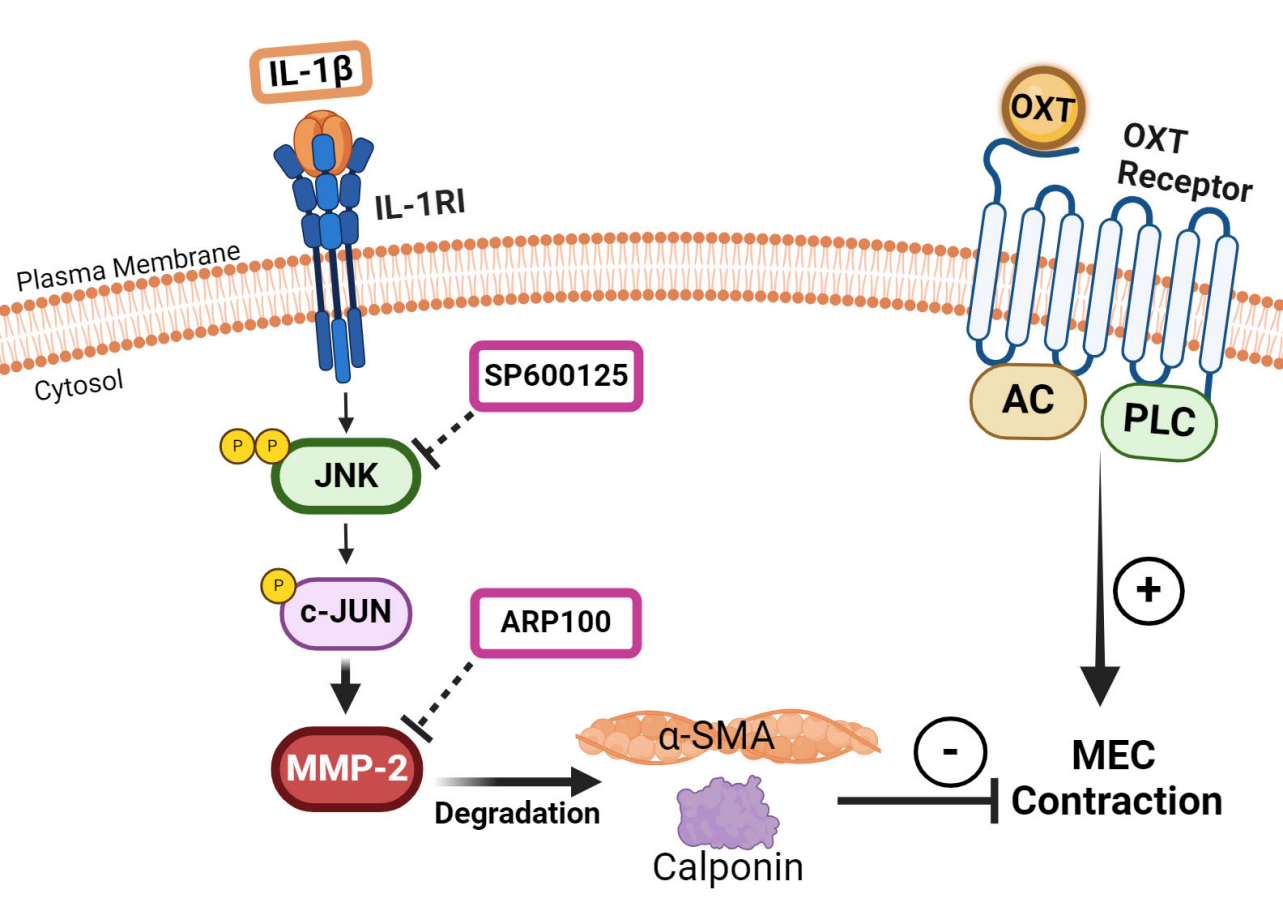
Schematic summary of our working hypothesis on the involvement of c-Jun N-terminal kinase (JNK) and matrix metalloproteinase-2 (MMP-2) in mediating the inhibitory effect of interleukin-1β (IL-1β) on oxytocin (OXT)-induced myoepithelial cell (MEC) contraction. Our data suggest that IL-1β activates JNK, which in turn activates MMP-2 in lacrimal gland MECs. Increased MMP-2 activity results in the degradation of α-smooth muscle actin (SMA) and calponin, which leads to an impaired MEC contraction in response to OXT stimulation. JNK, C-Jun N-terminal kinase; MMP-2, matrix metalloproteinase 2; AC, adenylate cyclase; PLC, phospholipase C.

Based on these reports, it is therefore plausible that, in our study on lacrimal gland MECs, IL-1β activates JNK, which in turn activates MMP-2 (**Figure 6**). An increased MMP-2 activity results in the degradation of SMA and calponin, which leads to an impaired MEC contraction in response to OXT stimulation (**Figure 6**). These *in vitro* findings might explain the beneficial effects of inhibiting JNK or MMP-2 in chronically inflamed lacrimal glands of Sjogren’s syndrome dry eye disease animal models, resulting in increased tear production (22, 30).

There are some limitations to our study, including the relatively small sample size and the inter-experiment variability inherent to the methods used. To remedy this latter limitation, we have measured the cell size and OXT-induced contraction from several cells within each individual experiment. Furthermore, the potential lack of specificity of the chemical inhibitors used in these studies should not be overlooked, and the development of more specific inhibitors or novel strategies to silence the JNK/MMP pathway is warranted.

In summary, our data establish a role for the JNK/MMP-2 pathways in IL-1β-induced alterations of the structure and function of lacrimal gland MECs, suggesting that targeting these pathways could be a viable option to alleviating dry eye disease induced by chronic inflammation, as occurs in Sjogren's syndrome.

## Data availability statement

The original contributions presented in the study are included in the article/**Supplementary Material**. Further inquiries can be directed to the corresponding author.

## Ethics statement

The animal study was approved by Tufts Medical Center Institutional Animal Care and Use Committee. The study was conducted in accordance with the local legislation and institutional requirements.

## Author contributions

JM: Data curation, Writing – review & editing, Formal Analysis, Investigation, Methodology, Resources. AG: Data curation, Formal Analysis, Investigation, Conceptualization, Writing – original draft. DT: Data curation, Investigation, Methodology, Writing – review & editing. SP: Data curation, Formal Analysis, Writing – review & editing. DZ: Data curation, Writing – review & editing, Conceptualization, Funding acquisition, Project administration, Supervision, Writing – original draft.
