# Supplementary material for: Interleukin-1β activates matrix metalloproteinase-2 to alter lacrimal gland myoepithelial cell structure and function
Source: Front Ophthalmol (Lausanne). 2024 Jun 6;4:1415002. doi: 10.3389/fopht.2024.1415002 (PMC11182216; doi:10.3389/fopht.2024.1415002)
Supplement: Supplementary file 1 [file DataSheet_1.docx]

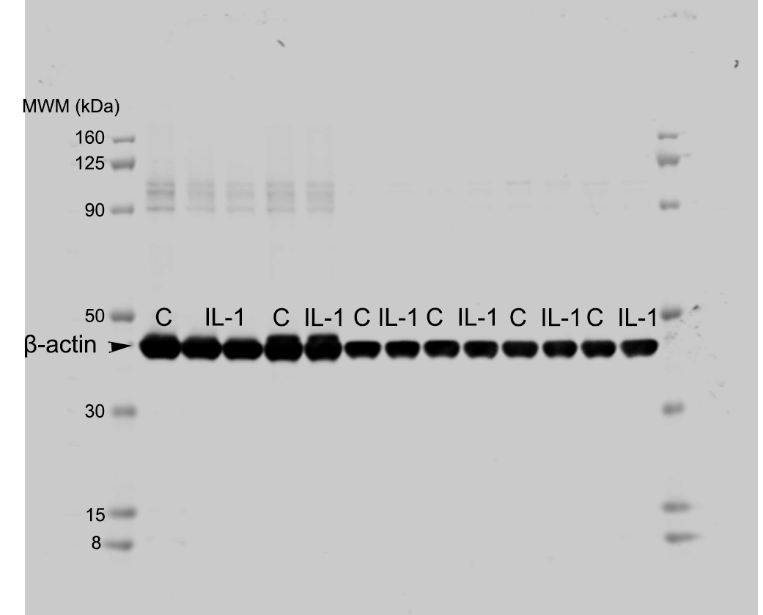




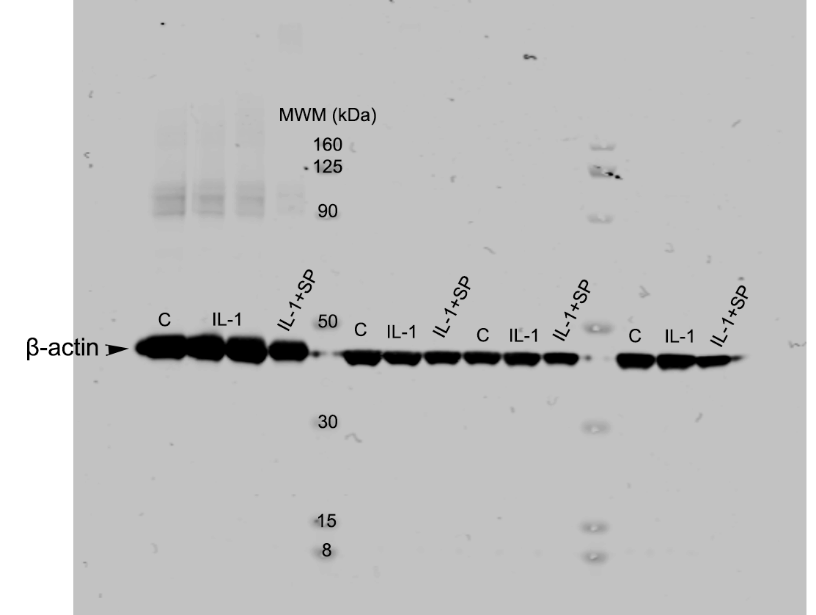


**Supplementary Figure 1.** Whole blots with molecular weight ladders depicting whole blot for pro-MMP2 and β-actin, used as a loading control.


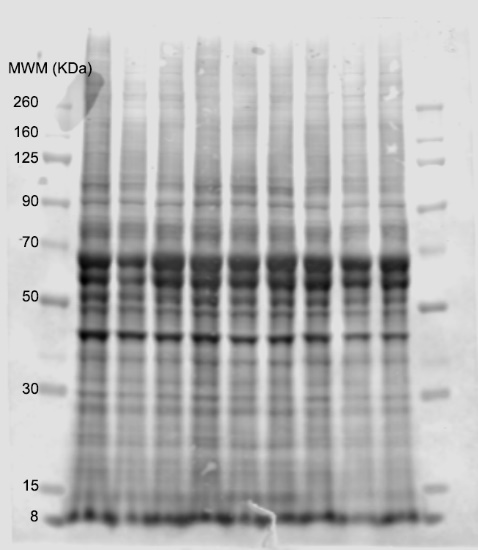


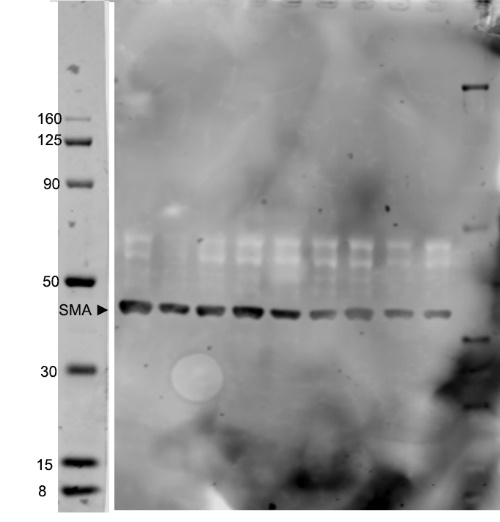


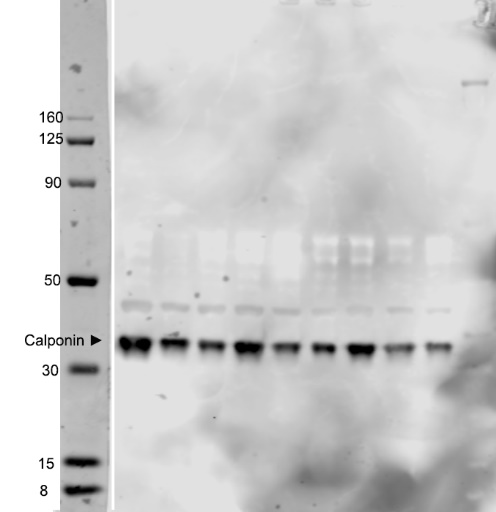


**Supplementary Figure 2.** Whole blots with molecular weight ladders depicting total protein stain, whole blot for SMA, and whole blot for calponin.


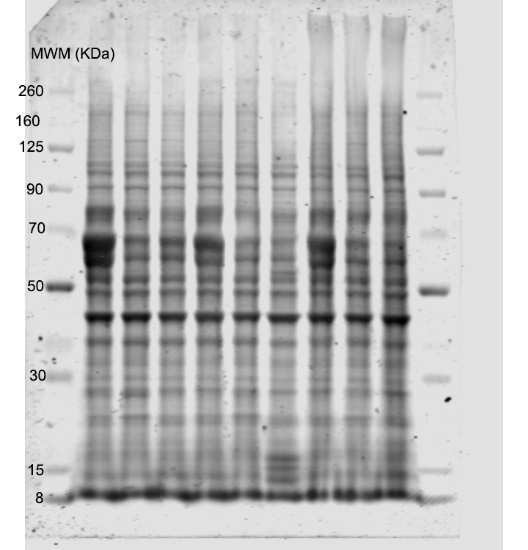


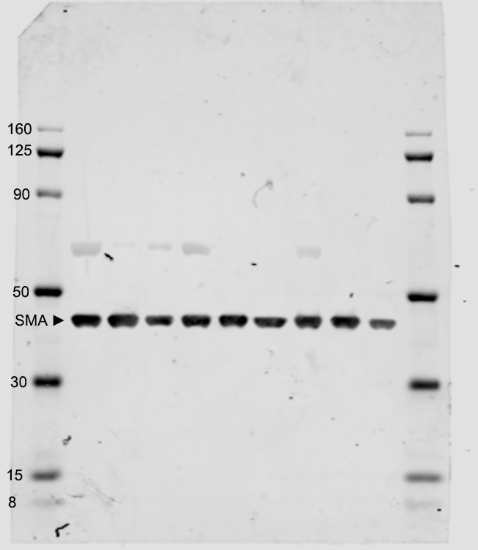


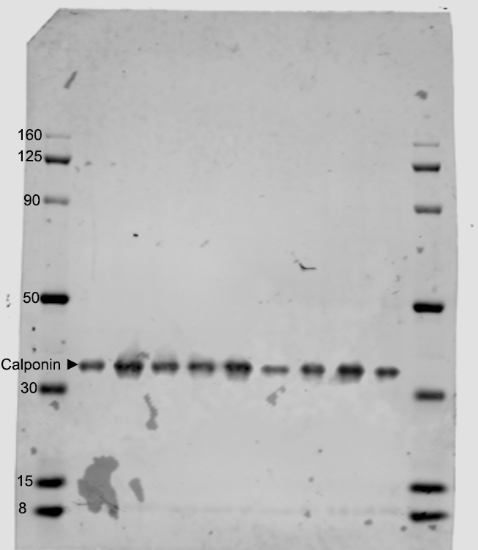


**Supplementary Figure 3.** Whole blots with molecular weight ladders depicting total protein stain, whole blot for SMA, and whole blot for calponin.


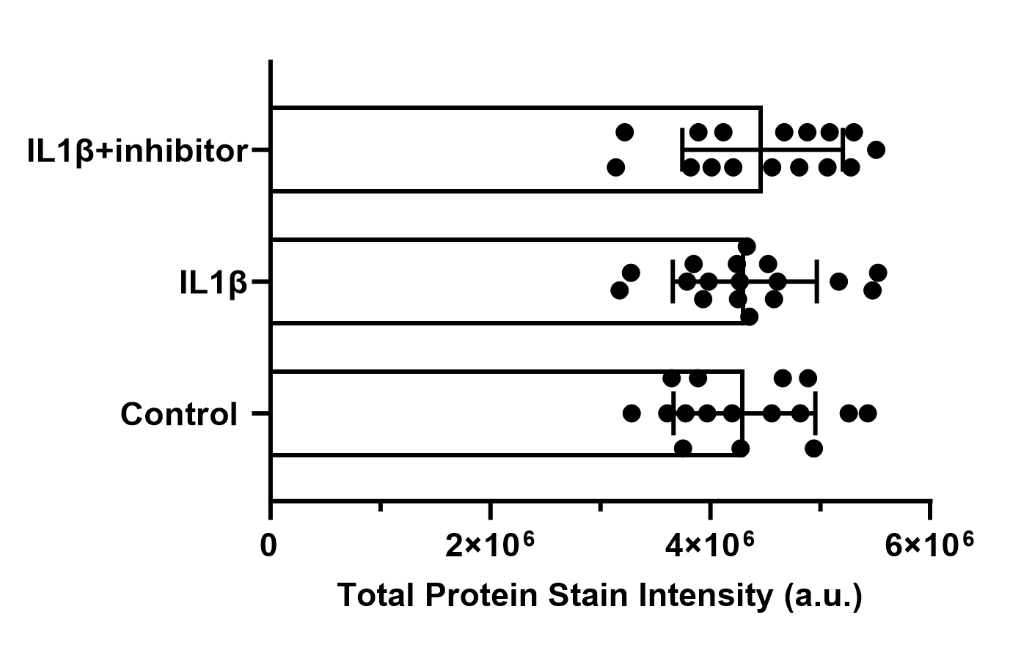


**Supplementary Figure 4.** Total protein stain intensity of samples from control untreated cells, IL-1β treated cells, and IL-1β + inhibitor (either SP600125 or ARP100) was quantified using ImageJ/Fiji software (ImageJ 1.54f, National Institutes of Health, USA). Data are presented as means ± SD, n=16-17.
